# Evaluation of Interventions to Improve Ventilation in Households to Reduce Risk for Transmission of Severe Acute Respiratory Syndrome Coronavirus 2

**DOI:** 10.20411/pai.v7i2.553

**Published:** 2022-12-10

**Authors:** Wilson Ha, Mitchell A. Stiefel, Jeremy R. Gries, Jennifer L. Cadnum, Maria M. Torres-Teran, Brigid M. Wilson, Curtis J. Donskey

**Affiliations:** 1 John Carroll University, University Heights, Ohio; 2 The Ohio State University, Columbus, Ohio; 3 St. Louis University, St. Louis, Ohio; 4 Research Service, Louis Stokes Cleveland VA Medical Center, Cleveland, Ohio; 5 Geriatric Research, Education, and Clinical Center, Louis Stokes Cleveland VA Medical Center, Cleveland, Ohio; 6 Case Western Reserve University School of Medicine, Cleveland, Ohio

**Keywords:** SARS-CoV-2, aerosol, ventilation, carbon dioxide, air cleaner

## Abstract

**Background:**

Inadequate ventilation may contribute to the high risk for household transmission of severe acute respiratory syndrome coronavirus 2 (SARS-CoV-2).

**Methods:**

We evaluated the effectiveness of several interventions recommended to improve ventilation in households. In 7 residential homes, carbon dioxide monitoring was conducted to assess ventilation in occupied open areas such as family rooms and in bedrooms and/or offices. Carbon dioxide levels above 800 parts per million (ppm) were considered an indicator of suboptimal ventilation for the number of people present. In 1 of the 7 homes, various interventions to improve ventilation or to filter air were assessed in a kitchen area by measuring clearance of aerosol particles produced using an aerosol-based spray system and carbon dioxide generated by cooking with a gas stove.

**Results:**

Carbon dioxide levels rose above 800 ppm in bedrooms and offices with 2 occupants when windows and doors were closed and in open areas during gatherings of 5 to 10 people; carbon dioxide levels decreased when windows or doors were opened. Clearance of carbon dioxide and aerosol particles significantly increased with interventions including running fans, operating portable air cleaners, and opening windows, particularly when there was a noticeable breeze or when a window fan was used to blow contaminated air outside.

**Conclusion:**

In households, several measures to improve ventilation or air filtration were effective in reducing carbon dioxide accumulation or enhancing clearance of carbon dioxide and aerosol particles. Studies are needed to determine if interventions to improve ventilation can reduce the risk for airborne transmission of SARS-CoV-2 in households.

## INTRODUCTION

Household contacts of individuals infected with severe acute respiratory syndrome coronavirus 2 (SARS-CoV-2) and other respiratory viruses are at risk of acquiring infection. [1–5]. The risk for transmission is relatively high in households because occupants are often in close contact for prolonged periods without wearing masks. The relatively poor quality of ventilation in households may also be an important contributor to the risk for transmission [6, 7]. It is recommended that homes receive a minimum of 0.35 outdoor air changes per hour (ACH) [7], whereas hospital rooms are required to have 8 or more air changes per hour [8]. In residential homes with central heating, ventilation, and air conditioning (HVAC) systems, air is typically recirculated without bringing in fresh outdoor air and without sufficient filtering to remove viral particles.

The Centers for Disease Control and Prevention (CDC) has recommended several measures that can be used to improve ventilation in households to reduce the risk for SARS-CoV-2 transmission [9, 10]. These measures include interventions to increase introduction of outdoor air such as opening windows and doors, using fans to move virus particles outdoors or to improve indoor airflow to reduce concentration of viral particles in particular areas, and improving filtration by using portable air cleaners or increasing the efficiency of the filters used in the HVAC system. However, there is limited evidence regarding the effectiveness of these interventions in household settings. Therefore, we conducted an evaluation of the impact of several interventions recommended to improve ventilation in households.

## METHODS

The study was approved as a quality improvement assessment by the Cleveland VA Medical Center's Research and Development Committee. In 7 residential homes, carbon dioxide monitoring was conducted to assess ventilation in occupied open areas such as family rooms and in bedrooms and/or offices. In 1 of the homes, ventilation was further assessed by measuring clearance of aerosol particles and carbon dioxide in a kitchen with different interventions to improve ventilation.

### Carbon dioxide monitoring

Carbon dioxide monitoring was conducted in 7 residential homes when people were present in open areas (eg, family rooms, dining rooms) and in bedrooms and/or offices. Carbon dioxide levels were measured using an IAQ-MAX CO2 Monitor and Data Logger (CO2Meter, Inc) that monitors carbon dioxide levels with a sampling time of 1.5 seconds. In open areas, monitoring was performed with 2 people present; in 3 homes monitoring was also performed in open areas during gatherings with 5 or more people present. In bedrooms or offices (N=8), monitoring was performed with 2 people present both with windows and doors closed and with windows and/or doors open. No identifiable information was collected from individuals present during monitoring. We recorded the number of people present during monitoring and factors affecting ventilation including whether doors or windows were open. Carbon dioxide levels were graphed over time.

The Centers for Disease Control and Prevention (CDC) has recommended that carbon dioxide readings above 800 ppm in buildings may be considered an indicator of suboptimal ventilation requiring intervention [6]. Peak levels above 800 ppm were therefore considered an indicator of suboptimal ventilation for the number of occupants present [6, 11].

### Clearance of carbon dioxide in a kitchen

One method that is commonly used to assess the adequacy of ventilation and the impact of ventilation interventions involves release of carbon dioxide with measurement of the rate of decay in carbon dioxide levels [12, 13]. For these assessments, carbon dioxide is often generated using dry ice or by mixing baking soda with vinegar [12]. The ventilation rate in air changes per hour can be estimated by measuring the time required for the carbon dioxide level to decrease by 63% [12, 13]. For the current investigation, carbon dioxide was generated through routine cooking using a gas stove. These experiments were included to provide a direct comparison of the impact of different interventions to improve ventilation.

In 1 of the 7 homes, we measured clearance of carbon dioxide in a kitchen to compare different interventions to improve ventilation. Carbon dioxide was generated during routine cooking with a gas stove for approximately 20 minutes. Levels of carbon dioxide were measured in a central location of the kitchen as previously described. Measurements were recorded at baseline and then at 10-minute intervals until carbon dioxide levels returned to approximately baseline levels or for up to 220 minutes.

The interventions included opening 2 windows after 20 minutes with or without a breeze, opening windows throughout including during cooking, and after 20 minutes with no breeze but with fans running, and keeping windows closed but with fans running. The fans that were used included the HVAC system fan, a ceiling fan on low speed, and the fan above the stove which was not ventilated outside. The assessments were repeated in triplicate for the baseline and for each intervention.

### Clearance of aerosol particles in a kitchen

Measurement of clearance of aerosol particles can be used to assess the adequacy of ventilation and the impact of air cleaning technologies [11, 14]. In the same kitchen used for the evaluation of carbon dioxide clearance, we examined the effect of interventions to improve ventilation and portable air cleaners on clearance of aerosol particles. For this assessment, a Preval sprayer (Nakoma Products, LLC) aerosol-based spray system was used to release ~6 mL of 5% NaCl over 15 seconds at a height of 6 feet in the center of the kitchen area. Based on particle count readings, the device disperses predominantly 0.3-5-μm droplets (authors' unpublished data). A 6-channel particle counter (Fluke 983, Fluke) was used to obtain particle count readings of 1 to 10 μm diameter particles at baseline and at 5-minute intervals until particle counts returned to approximately baseline levels or for up to 60 minutes. Readings were obtained with all windows closed and with no fans operating (control) and with several interventions intended to improve ventilation.

The interventions included opening 2 windows with or without a breeze, opening windows with no breeze but with a window fan in 1 window blowing to the outside, windows closed but with fans running, and windows closed with a portable air cleaner running in the central area of the kitchen. The fans that were used included the HVAC system fan, a ceiling fan on low speed, and the fan above the stove which was not ventilated outside. Two air cleaners were tested including a commercial Germ Guardian 5-in-1 28” Pet Pure Air Purifier with HEPA Filter, UV-C Sanitizer and Odor Reduction (Guardian Technologies, LLC) intended for use in rooms up to 117.6 m^2^, and a do-it-yourself air cleaner with minimum efficiency reporting value (MERV)-13 filters constructed according to instructions available online [15]. A box is made with 4 MERV-13 filters of 20” (sides of the box), a 20” box fan (top of the box), and a cardboard bottom. The airflow rates of the Germ Guardian and do-it-yourself air cleaner with MERV-13 filters were 11.63 m^3^/min and 34.16 m^3^/min, respectively [14]. The assessments were repeated in triplicate for the baseline and for each intervention.

### Data analysis

For the carbon dioxide monitoring, we evaluated conditions resulting in carbon dioxide levels above 800 ppm without performing statistical comparisons. For the clearance of carbon dioxide and aerosol particles, starting at 20 minutes for carbon dioxide and 5 minutes for aerosol particles, decay curves were estimated in a single nonlinear least squares model for each intervention relative to the non-intervention curve of windows closed throughout. We compared the levels at which carbon dioxide and particles levels stabilized and the peak particle counts. The times at which predicted aerosol particles fell below 5,000 particles were calculated. All analyses were performed in R Version 4.3.1 software (The R Foundation for Statistical Computing, Vienna, Austria).

## RESULTS

### Carbon dioxide monitoring

**Figure 1.**
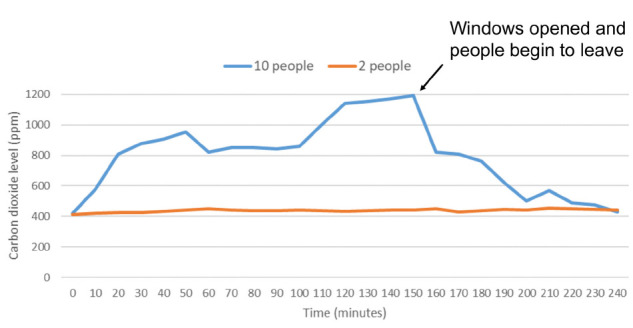
**Family room.** Carbon dioxide levels in parts per million (ppm) in a family room (volume, 50.7 m^3^) open to other areas in the house with either 2 or 10 people present. The windows were closed except as indicated by the arrow when 10 people were present. Peak levels of carbon dioxide above 800 parts per million (ppm) were considered an indicator of suboptimal ventilation for the number of occupants present.

In the 7 residential homes, carbon dioxide levels in open areas such as family rooms remained below 800 ppm when 2 people were present. In 3 of the homes, carbon dioxide levels were monitored in open areas during gatherings of 5 to 10 people. For each of these gatherings, carbon dioxide levels rose above 800 ppm during the gathering. Figure 1 shows carbon dioxide levels in 1 family room with 2 people present and during a gathering with 10 people present; the initial readings were recorded at the same time the people entered the house. The carbon dioxide level rose above 800 ppm when 10 people were present and decreased rapidly when the windows and doors were opened, and the number of occupants decreased to 2 over the next 30 minutes. In another gathering including 4 to 8 people and 3 dogs, the carbon dioxide level peaked at greater than 1,400 ppm.

**Figure 2.**
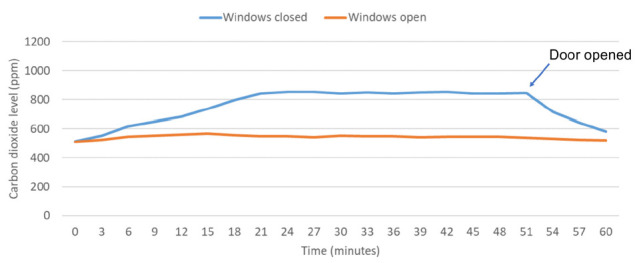
**Study with 2 occupants.** Carbon dioxide levels in parts per million (ppm) in a study (volume, 30.0 m^3^) with 2 people present with the windows open and door closed and with the windows and door closed followed by opening the door after 50 minutes. Peak levels of carbon dioxide above 800 parts per million (ppm) were considered an indicator of suboptimal ventilation for the number of occupants present.

Carbon dioxide levels were measured in 7 bedrooms or offices with 2 people present. In 6 of the 7 (86%) bedrooms or offices, carbon dioxide levels rose above 800 ppm when windows and doors were closed. The carbon dioxide levels decreased in each of the rooms after windows or doors were opened. Figure 2 shows carbon dioxide levels in a typical small room with 2 occupants with the windows open and door closed as well as with the windows and door closed followed by opening the door after 50 minutes; the initial readings were recorded at the time the people entered the room. The HVAC system fan was operating on continuous mode during these evaluations.

### Clearance of carbon dioxide

**Figure 3.**
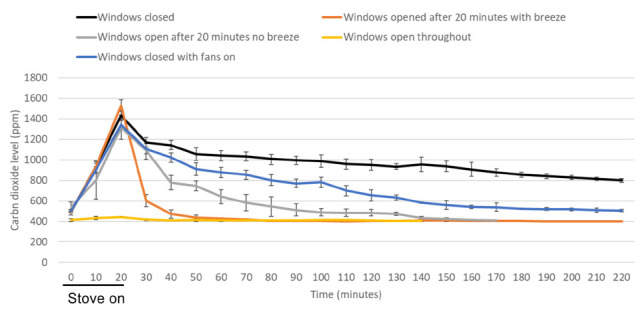
**Clearance of carbon dioxide generated by cooking with a gas stove.** Clearance of carbon dioxide generated by cooking with a gas stove in a kitchen (volume, 43.5 m^3^) with windows closed versus with several different interventions to improve ventilation. Fans included an overhead ceiling fan, stove fan without external ventilation, and the central air conditioning fan on in continuous mode. Average results for 3 experiments are shown. Error bars represent standard deviation.

Figure 3 shows clearance of carbon dioxide over time in a kitchen. Carbon dioxide levels rose above 800 ppm after 20 minutes with the stove on in all the assessments except when the windows were open throughout the monitoring period. With the windows closed and with no fans operating (control), the carbon dioxide level remained above 800 ppm until 220 minutes after the start of monitoring. Each of the interventions resulted in decay to significantly lower carbon dioxide levels (*P*<0.001). The rate of carbon dioxide decay was significantly faster with windows open with a breeze versus with no breeze or with windows closed but with fans operating (*P*<0.001). The time to carbon dioxide less than 800 ppm was 10 minutes with the windows open with a breeze present versus 40 minutes when no breeze was present, and 90 minutes with windows closed but with fans on.

### Clearance of aerosol particles

**Figure 4.**
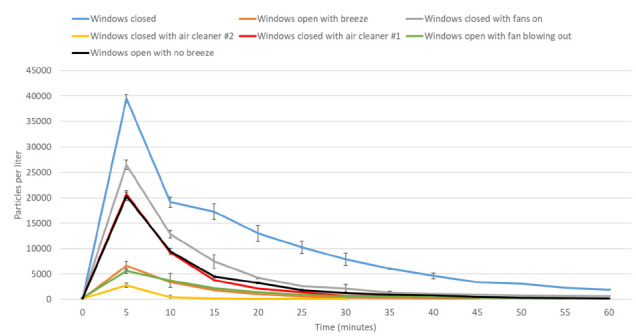
**Clearance of 5% sodium chloride solution aerosol particles (1 to 10 μm diameter) in a kitchen with windows closed versus with several different interventions to improve ventilation.** Fans included an overhead ceiling fan, stove fan without external ventilation, and the central air conditioning fan on in continuous mode. The aerosol particles were released over 15 seconds. Average results for 3 experiments are shown. Error bars represent standard deviation.

Figure 4 shows clearance of aerosol particles over time in a kitchen. With the windows closed with no fans operating (control), the particle counts peaked at a mean of 39,490 and decreased to less than 5,000 after 40 minutes and to a mean of 1,964 by 60 minutes. In comparison to the control, each of the interventions resulted in lower peak particle counts at 5 minutes and decay to significantly lower particle counts (*P*<0.001). The estimated number of minutes to a particle count of less than 5,000 were control (37), windows closed with fans on (19), windows open with no breeze (15), windows closed with air cleaner #1 (14), windows open with breeze (8), and windows open with fan blowing out (7). The mean particle counts did not rise above 5,000 when the windows were closed with air cleaner #2 operating.

## DISCUSSION

The CDC has provided guidance on several measures that can be taken to improve ventilation in households to reduce the risk for SARS-CoV-2 transmission [9, 10]. However, little real-world evidence has been available on the efficacy of these interventions in typical residential households. In the current study, we demonstrated using carbon dioxide monitoring that ventilation may be suboptimal in rooms in houses with the doors closed and in open family rooms during gatherings with large numbers of people. In these areas, opening windows or doors prevented increases in carbon dioxide levels or resulted in rapid reductions in elevated carbon dioxide levels.

In 1 household, clearance of carbon dioxide and aerosol particles introduced into a kitchen area occurred slowly when the windows were closed (220 minutes for carbon dioxide to decrease from 1,462 to less than 800 ppm and 40 minutes for particles to decrease from a mean of 39,490 to less than 5,000. Several ventilation interventions recommended by the CDC were effective in enhancing clearance of aerosol particles and carbon dioxide [9, 10]. Operating fans including the HVAC system fan, ceiling fan, and above-stove fan resulted in a relatively modest enhancement in clearance of carbon dioxide and particles. Opening windows was more effective in enhancing clearance, particularly if there was a noticeable breeze or if a window fan was used to blow contaminated air outside. Portable air cleaners were effective in enhancing particle clearance when the windows remained closed. Notably, the do-it-yourself air cleaner with MERV-13 filters had a much higher airflow rate than the commercial air cleaner and was substantially more effective in rapidly reducing aerosol particles.

Our study has some limitations. We conducted testing in a small number of households and did not have information on the air changes per hour for the houses. However, results were similar in each of the houses. We did not examine the impact of improving the efficiency of HVAC system filters on clearance of aerosol particles [9, 10]. Elevated carbon dioxide levels have not been directly linked to SARS-CoV-2 transmission risk. However, poorly ventilated indoor spaces are generally considered high-risk areas [6, 7, 16–18]. We did not examine the potential for fans to increase transmission risk if they direct airflow from an infected source patient to susceptible individuals. Previous studies have suggested that patterns of airflow might have contributed to long-distance transmission of large and small droplets containing SARS-CoV-2 in settings such as restaurants, patient transport vans, and double-occupancy patient rooms [17, 19, 20]. Our investigation did not address measures other than ventilation that might affect transmission risk in households (eg, use of facemasks and physical distancing). Finally, we found that air cleaners were effective in reducing aerosol particles, but it is not known if these devices are effective in reducing household transmission of airborne pathogens. A recent systematic review did not identify any studies that investigated the effects of air filters on the incidence of respiratory infections [21].

In conclusion, several measures recommended to improve ventilation or air filtration in households were effective in reducing carbon dioxide accumulation or enhancing clearance of carbon dioxide and aerosol particles. These measures could potentially be useful to reduce the risk for airborne transmission of SARS-CoV-2 and other respiratory viruses if household members or visitors are infected. Studies are needed to test the effectiveness of interventions to improve ventilation for prevention of transmission of respiratory viruses in households.
